# Flyway population increase and emergence of new wintering grounds with climate change in an Arctic-breeding goose

**DOI:** 10.1038/s41598-026-40447-0

**Published:** 2026-03-03

**Authors:** Péter Gyüre, Szabolcs Lengyel

**Affiliations:** 1 Faculty of Agricultural and Food Sciences and Environmental Management, Institute of Animal Science, Biotechnology and Nature, University of Debrecen, Böszörményi út 138, Debrecen, 4032 Hungary; 2 HUN-REN Centre for Ecological Research, Institute of Aquatic Ecology, Conservation Ecology Research Group, Bem tér 18/c, Debrecen, 4026 Hungary; 3 Biodiversity, Climate Change and Water Management Competence Centre, University of Debrecen, Vezér u. 37, Debrecen, 4032 Hungary

**Keywords:** Climate change, Migration, Overwintering, Phenology, Game or quarry species, Staging, Stopover, Waterfowl, Wildlife management, Climate sciences, Ecology, Ecology, Environmental sciences

## Abstract

Changes in climate, land cover, hunting disturbance, and food availability are often cited as drivers of bird migratory patterns. We examined long-term changes in the Pannonic flyway population of the Arctic-breeding Greater White-fronted Goose (GWfG, *Anser albifrons*) in regular counts conducted in Hortobágy National Park, eastern Hungary, between 1989 and 2019. During 31 years, GWfG counts increased from fewer than 2,000 to more than 30,000 individuals, with a stronger increase in spring than in autumn. The rate of increase exceeded that of the entire Pannonic flyway population. Spring counts rose more rapidly in the early years (1989–1997), whereas autumn counts increased faster more recently (2007–2019). Overwintering began in the mid-2000s, and winter counts have increased sharply since 2007. Mean temperature influenced GWfG counts positively in winter and negatively in spring, while the number of frost days in winter and snowy days in spring had negative effects. GWfG counts decreased less from December to January when temperatures did not drop or increased. These results indicate that milder winters have played a major role in the long-term increase and appearance of overwintering. In addition, small changes in land cover and slight increases in hunting disturbance may have also contributed to the increase of the GWfG counts at the study site, whereas food availability on nearby croplands declined and may have had an opposite effect. Our study shows that the disproportionate increase in staging and overwintering GWfG is primarily driven by climate change, especially by the rising frequency of unusually mild winters.

## Introduction

Climate change is one of the major drivers of the decline of biodiversity^1^ and it can affect migratory birds in time (phenology), space and numbers^2^. Phenological changes include the advancement or delay of events in the annual cycle such as earlier spring arrival to the breeding grounds, earlier start of breeding, longer breeding season, earlier or later autumn departure, earlier or later arrival to stopover sites and wintering grounds, and longer time periods spent in stopover sites and/or wintering grounds^3–5^. The spatial changes include the rearrangement of migration flyways and the use of different stopover sites and wintering grounds. Older flyways may be used by progressively fewer birds or completely displaced, and new flyways can form, along with changes in the stopover sites and wintering grounds used, often resulting in shorter migration distances^6,2,7^. In several Northern Hemisphere species, northward shifts of stopover sites were found^8–10^. Finally, changes at the breeding, stopover and wintering grounds can result in population increases or decreases. Arctic-breeding geese appear to benefit from the earlier onset of spring and longer breeding periods^5^, and the wider availability of intensive winter croplands during migration^11^. All but two of 21 Arctic goose populations with good data show positive long-term trends, and 18 populations have increased considerably since the early 1990s^12^. Larger population sizes mean wider migration fronts, more intense use of stopover sites and wintering grounds, and increased goose numbers often represent challenges to agriculture, conservation, management and monitoring in Europe and North America^3,13^.

The greater white-fronted goose (*Anser albifrons*, GWfG hereafter) is one of the most widespread Arctic-breeding migratory goose species in the Western Palearctic. The Eurasian populations of the species have generally increased in the last three decades^12,14,15^. The two westernmost Eurasian populations of the species form two distinct flyway populations: the North Sea and the Pannonic (a.k.a. Western Siberian / Central European) populations^16^. The larger North Sea population (c. 1.2 million individuals) breeds west of the Ural Mountains, has lower breeding success and is stable in size, and migrates westward along the Arctic, Baltic and North Sea coasts to wintering areas in northern Germany and the Netherlands^17^. The smaller Pannonic population (c. 140,000 individuals) breeds east of the Urals, has a higher breeding success and has been increasing since 1986, and migrates southward east of the Urals to stopover sites in north-western Kazakhstan^18^, then continues westward and reaches wintering areas in the Carpathian Basin from the east^17^. The Pannonic flyway population increased annually by 7.5% between 1988 and 2012, and by 6.5% between 2003 and 2012^14^. While the timing of migration, the use of stopover sites and wintering grounds, and the size of the North Sea population are rather well-known, much less is known on the migration or the size of the Pannonic population^12,14^. For instance, it is not known whether recent increases in goose numbers in north-western Europe are the result of changes in survival and breeding success within a geographically defined section of the North Sea population, or due to shifts in the distribution of individuals among stopover sites and wintering grounds. Specifically, a climate change-driven northward shift of migration flyways could mean that GWfG that formerly wintered in Hungary no longer do so but migrate directly to the Netherlands via more northerly stopover sites such as south-west Poland^19^.

The Hortobágy region in Eastern Hungary has long been a major centre of staging and wintering for wild geese in Europe. In the early 20th century, hunters were attracted from all over Europe by the enormous abundance of goose flocks migrating through the region^20^. Up to 500,000 geese were reported to stage regularly in Hungary in the first half of the 20th century, and GWfG was by far the most abundant species^21,22^. The number of GWfG staging in Hungary decreased from the 1950s until the 1980s, likely due to hunting and disturbance at the roosting areas^23^. The staging/wintering population of GWfG in Hungary has increased since the 1980s, with some sudden increases in peak numbers, e.g. 165,771 individuals counted in February 1992^24^. Between 1996 and 2006, the number of GWfG increased from 67,521 to between 73,668 and 126,811 individuals depending on the year, corresponding to a trend of + 73% in only 10 years^21^. The centre of GWfG migration is the Great Plains area of eastern Hungary, however, the numbers of GWfG have also increased in the western part of Hungary^21^.

The aim of this work is to describe recent changes in the number of GWfG staging and overwintering in the Hortobágy region, the most important stopover and wintering area in Hungary, an understudied part of the range of this species. We present a long-term, 31-year dataset collected on the number of GWfG in systematic counts of individuals in Hortobágy National Park and specifically evaluate whether changes in climate, land cover, food availability, or hunting disturbance affected the staging and overwintering of GWfG. We specifically tested four hypotheses. First, we tested whether climate change, especially in terms of warmer temperatures and milder winters, influenced the number of GWfG staging or overwintering in the study region. Second, we hypothesised that long-term land cover change resulted in an increased availability of three resources used by GWfG during staging or overwintering: (i) grasslands as the primary food source, (ii) croplands as the secondary, often more profitable food source, and (iii) inland marshes and waters as the areas used for night roosting. Third, we expected that the availability of stubbles of crops preferred for feeding by GWfG (maize and wheat) increased during the study period and we predicted that GWfG numbers would follow this increase. Finally, we studied whether increasing hunting pressure and/or disturbance in nearby areas could explain the increase of GWfG numbers in the study area.

## Methods

### Study area

**Fig. 1:**
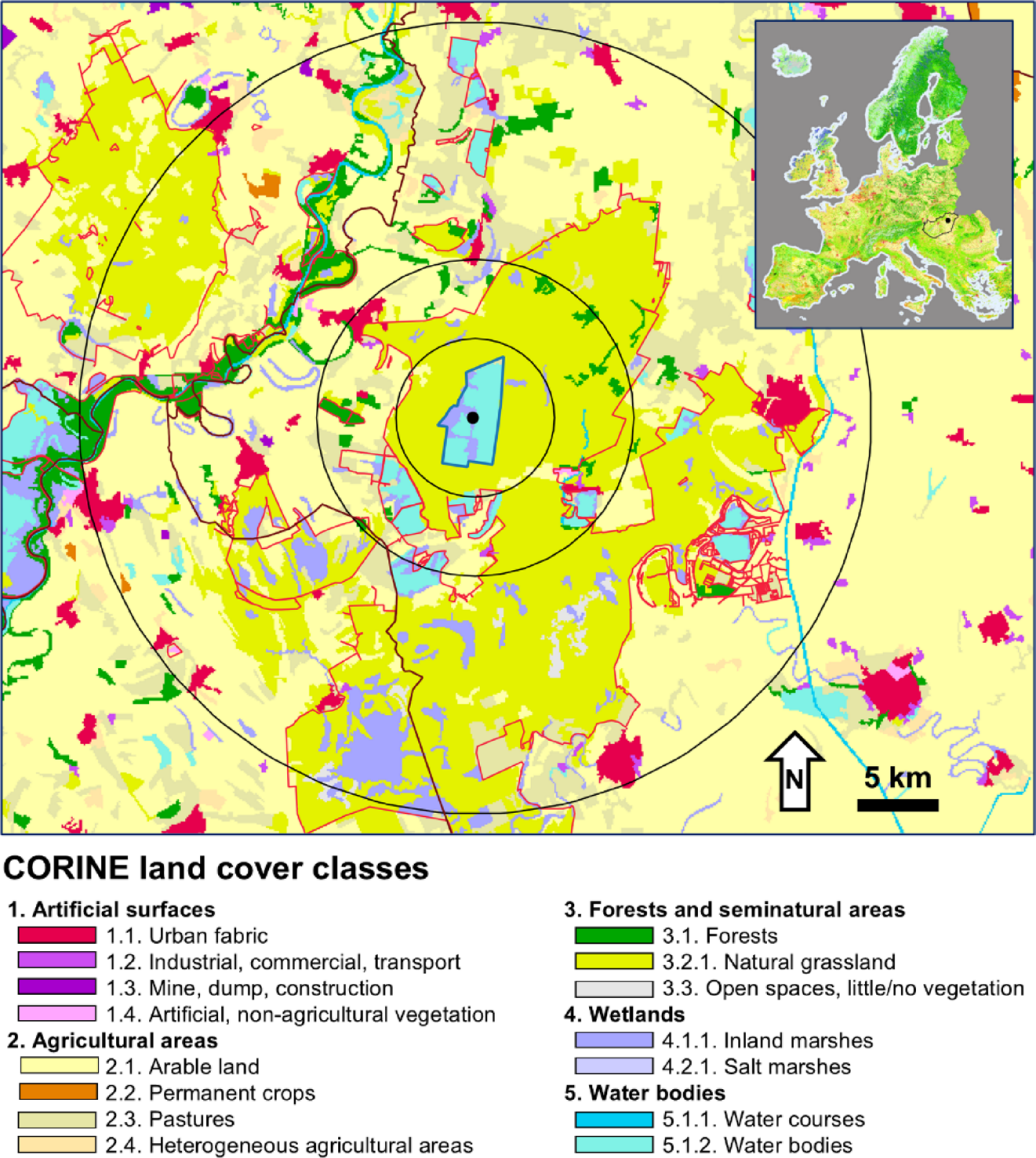
CORINE land cover map of the study area (year 2018), the location of the Central Fishponds (black dot) and 5-km, 10-km and 20-km buffers (black circles) in Hortobágy National Park, E Hungary. Red lines indicate protected area borders and brown lines indicate county borders. The map was created in QGIS (version 3.40.7) (QGIS Development Team^27^).

Hortobágy National Park (82,000 hectares) preserves one of the largest contiguous grassland-wetland complexes in Europe and one of the westernmost occurrences of open Eurasian steppes in the east-central part of the Carpathian Basin (Fig. 1). The Hortobágy landscape was shaped by floods of river Tisza, grazing by large herbivores and fire. Alkali soils have been present in the region at least since the last glacial cycle and the first evidence for the presence of pastoralist cultures dates to 3300 BC^25,26^. The park area, a mosaic of dry loess grasslands, alkali steppes, meadows and marshes, as well as fishponds, pastures and farms, is a cultural World Heritage Site and its wetlands are designated as sites of international importance under the Ramsar Convention. The park area is surrounded by arable land (Fig. 1), mostly by intensively cultivated croplands (wheat, maize, sunflower).

Hortobágy National Park is the most important staging area of GWfG in Hungary both based on the number of geese regularly observed and based on its geographic position as it is the first and largest grassland/wetland area encountered by geese flying through the Carpathian Mountains from the east. The study area comprised the main fishpond system in the central part of the park (Hortobágy Central Fishponds, 1817 ha) and the surrounding grasslands and croplands (47°35′40″ N 21°9′24″ E) (Fig. 1).

### Field methods

Most GWfG arrive to Hortobágy in October, the autumn peak is in November, the winter minimum is in January, and the spring peak is in February and March; and by April, GWfG usually leave the area^24^. We designated seasons as follows: autumn lasted from Sep 15 to Nov 30, winter from Dec 1 to Jan 31, and spring from Feb 1 to April 15. In the Hortobágy region, GWfG use the natural grasslands and surrounding croplands as daytime feeding areas and artificial fishponds and natural marshlands as night roosting areas. Goose monitoring was conducted by PG once every two weeks between 1 Jan 1989 and 31 December 2019 (total 378 goose-counting occasions in 31 years). At each monitoring occasion, geese were counted in the morning as they departed from the night roosting area to the feeding areas and at midday as they returned from the feeding areas for their midday resting. Binoculars and spotting scopes were used to count geese on the fishponds from observation towers^28^.

### Response variables

To analyse long-term trends, we first took the peak counts, i.e., the maximum number of GWfG counted on any day within a month (monthly peak) or a season (autumn, winter, spring; seasonal peak) as the dependent variable. We used peak counts to demonstrate long-term trends to reduce the non-independence of datapoints arising from potential double-counting of individuals. However, because peak counts may not reflect the total migration population due to differences in phenology between years and within seasons, we also calculated the number of goose-days for each month, season and year and used the number of goose-days in the relevant analyses. Because peak counts and number of goose-days varied considerably and were not normally distributed, we log-transformed them for all statistical analyses.

In analyses of the effect of climate change, we also used the original biweekly counts and number of goose-days as data on weather were available for every day in the study period. In analyses of land cover change, food availability and hunting disturbance, we used either the spring or the autumn peak counts or the spring or autumn sum of the number of goose-days because the data on these factors were available only for single years.

### Trend analyses

We used linear regression of the log-transformed peak counts on year to test long-term trends in GWfG numbers. To test whether the rate of change in GWfG counts differed in time periods, we calculated break-point (or piecewise) regressions for the spring and the autumn counts of GWfG using the ’segmented’ package of R, which automatically identifies break-points^29^. For visualization, we also fit smoothed lines to the spring and autumn counts by using the ‘loess.as’ function of the ‘fANCOVA’ package in R, which automatically selects an optimal value for the smoothing parameter^30^. Finally, to allow the comparison of the rate of increase of the total flyway population and the flyway population counted in the study site, we used the January counts between 1988 and 2012 or between 2003 and 2012 because the estimates on the total flyway population are also based on the January counts in these periods^14^.

### Climate change analyses

To test potential changes due to climate change, we modelled temporal and weather-related variation in biweekly GWfG counts and number of goose-days from October to March over 31 years using a generalized additive mixed model (GAMM) as implemented in the ‘bam’ function of the ‘mgcv’ package of R^31^. We obtained daily data on air temperature, precipitation and snow cover for the Debrecen station (47.57° N 21.58° E) located c. 30 km east from the study area from the Hungarian Meteorological Service (https://www.met.hu/eghajlat/magyarorszag_eghajlata/eghajlati_adatsorok/Debrecen). From these data, we calculated mean temperature (TEMP), sum of precipitation (PREC), number of frost days (minimum daily temperature < 0 °C, FROSTD) and number of days with snow cover (SNOWD) for each biweekly period between October 1 and March 31 in each year. The model was specified as:

$$\begin{aligned}Y&=\text{te(YEAR, WEEK, bs}=c(\mathrm{tp}\mathrm{,cc))}+s\left(\mathrm{TEMP}\right)+s\left(\mathrm{PREC}\right)+s\left(\mathrm{FROSTD}\right)\nonumber\\ &\quad +\mathrm{s}\left(\mathrm{S}\mathrm{N}\mathrm{O}\mathrm{W}\mathrm{D}\right)+\mathrm{s}(\mathrm{O}\mathrm{B}\mathrm{S}\_\mathrm{I}\mathrm{D},\:\mathrm{b}\mathrm{s}=\mathrm{c}\mathrm{r}),\end{aligned}$$

where *Y* was the biweekly GWfG counts or number of goose-days. The tensor product smooth te(YEAR, WEEK) allowed simultaneous modelling of long-term, non-linear between-year effects (YEAR, bs = “tp”) and cyclic within-season effects (WEEK, i.e. ID of biweekly period), and also allowed the shape of the seasonal pattern to vary across the years. The cyclic pattern was made possible by the specification of a cyclic cubic spline basis for WEEK (bs = “cc”). The weather covariates (TEMP, PREC, FROSTD, SNOWD) were included as separate smooth functions to allow them to vary by season (autumn: Oct-Nov, winter: Dec-Jan, spring: Feb-March). We standardised all continuous covariates (mean = 0, S.D. = 1) prior to modelling to facilitate the comparison of effect sizes. We accounted for overdispersion in the count data by fitting GAMMs with negative binomial error distribution with a log-link. We also accounted for temporal autocorrelation by adding the term s(OBS_ID, bs = “cr”), where OBS_ID was a sequential observation ID, and which fits a cubic line regression, i.e., a smooth curve over time that explains variation not captured by the tensor product smooth for the long-term trends and cyclic seasonal structure. The bam() function used deals with autocorrelation inside the linear predictor, not in the error term, and smooths of time should be used instead of temporal autocorrelation error structures unless there is a very strong reason^31^.

Model selection and diagnostics followed standard procedures, including visual inspection of residuals, checks for concurvity, and approximate significance testing of smooth terms using functions ‘summary.gam’ and ‘gam.check’. We estimated the final model using fast restricted maximum likelihood (fREML) to ensure robust smoothing parameter selection. Finally, to study the effect of weather on overwintering, we analysed the changes in goose numbers from December to the following January as a function of changes in weather variables.

### Land cover change

To quantify changes in land cover in the 30-year study period (1989–2019), we used the land cover change data from the CORINE Land Cover (CLC) dataset^32^. Land cover change data were available from four periods (1990 to 2000, 2000 to 2006, 2006 to 2012, 2012 to 2018) (CLC version 2020, download date 2025.06.02.). We calculated the change in three land cover types that are the most important to GWfG: (i) cropland areas as potential feeding sites, for which land cover type ’non-irrigated arable land’ was used for analysis, (ii) grassland areas as potential feeding sites for which categories ’natural grasslands’ and ’pastures’ were combined, and (iii) wetland areas as potential resting and roosting sites (’inland marshes’ and ’water bodies’ combined).

Because GWfG use both areas close to and far from the study fishpond system, we studied land cover change in three buffers of different size: 5, 10, and 20 km measured from the centroid of the Central Fishponds. The 5-km buffer corresponded to areas within the national park, the 10-km buffer included some neighbouring areas outside the park, whereas the 20-km buffer included large areas outside the national park (Fig. 1). In total, we calculated land cover change in four periods and three buffer sizes, resulting in *n* = 12 estimates. We used QGIS version 3.40 and the ETRS/LAEA geographic coordinate system for all area calculations.

To test the potential effect of land cover change on goose counts and number of goose-days, we calculated the estimated trend of the migrating GWfG population separately for autumn and spring within each of the four CLC periods as the response variable. To estimate the trend, we calculated the non-parametric Spearman’s correlation coefficient between either the annual maximum number of geese counted or the total number of goose-days in the autumn or the spring and the years within each period. We then tested the relationship between the trend estimate and the change in land cover by using linear regressions within each buffer in each period (*n* = 12 regression models per dependent variable, i.e., 24 models total).

### Food availability

To characterise food availability, we obtained data on the area sown with maize and wheat in Hajdú-Bihar county from the Hungarian Central Statistical Office (https://www.ksh.hu/stadat_files/mez/hu/mez0071.html). While the study area is confined to a small part of the county, the long-term (20 + years) trend in the area sown with these two crops near Hortobágy National Park is not likely to differ from the trend in the rest of the county. Both crops are frequently used by GWfG for feeding in the Hortobágy region. Wheat fields are used mostly in the spring migration whereas maize fields are mostly used in the autumn, when geese and other migrating species such as Common Cranes typically feed on maize stubble left over after harvest^33^. We tested the potential effect of the availability of food on croplands on goose counts and number of goose-days by using linear regression models of goose counts or goose-days in the autumn or the spring against the area sown with wheat and the area sown with maize. While maize is more important to geese in the autumn and wheat is more important for them in the spring, we performed analyses with both dependent variables in all potential combinations (total eight linear regression models).

### Hunting disturbance

Since the foundation of Hortobágy National Park in 1973, goose-hunting has been banned within the national park area (Fig. 1), whereas duck-hunting occurred until 1991. Goose-hunting is allowed in areas outside the national park boundaries in December and January^34^. To assess the potential impact of disturbance from hunting activities on GWfG numbers, we used data on hunting bag of all goose species as a proxy for disturbance. We obtained data on annual goose hunting bag in Hajdú-Bihar county (where the study area is located) and four neighbouring counties with ample goose habitats (Békés, Borsod-Abaúj-Zemplén, Heves, and Jász-Nagykun-Szolnok) from 31 years between 1989 and 2019 from the National Game Management Database of Hungary (http://www.ova.info.hu/index-en.html). Assuming that hunting bag data reflects disturbance outside the national park boundaries, we then used these data to study whether and how hunting disturbance changed by years and whether increases in hunting bag in neighbouring counties were associated with increases in GWfG counts or number of goose-days at the study site. Because hunting bag data are available as sums of the hunting season (Dec-Jan), we also used the sum of GWfG counts and number of goose-days from these two months. We used linear regression models to test the relationship between (i) goose hunting bag and year, and mean GWfG counts in winter and year, and (ii) goose hunting bag and GWfG counts in winter. In the first analysis, we also tested the differences in slope between the two relationships and in both analyses, we also tested the quadratic term to allow for non-linear effects.

## Results

### Long-term trends in migrating goose numbers

**Fig. 2:**
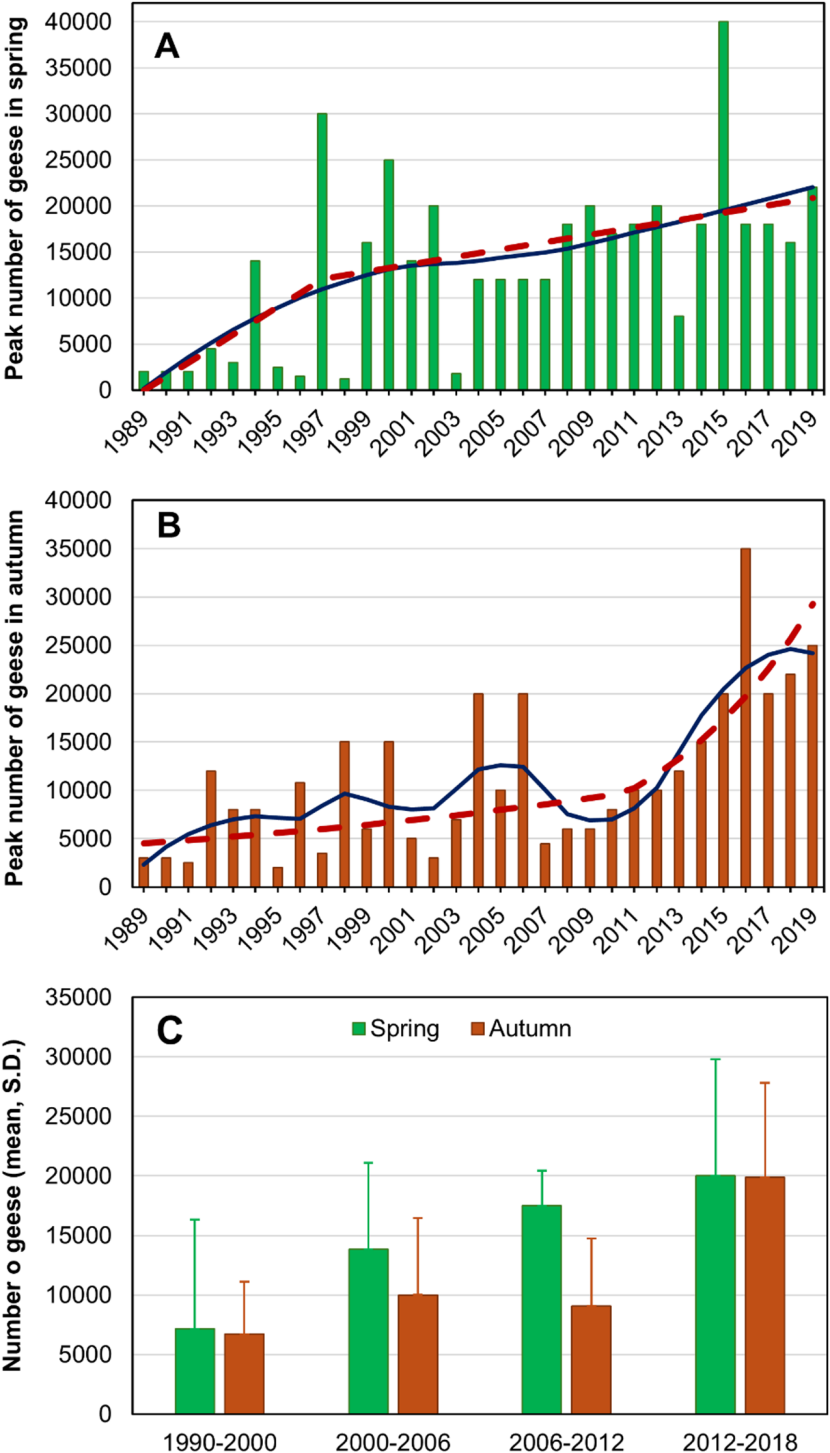
Peak counts of the greater white-fronted goose (*Anser albifrons*) in the spring (March, **A**) and autumn (November, **B**), and mean ± S.D. annual peak counts in the four study periods (**C**) in the Hortobágy Central Fishponds in 1989–2019. Red dashed lines indicate values predicted by break-point regression and blue lines indicate values predicted by LOESS regression.

Throughout the 31-year study period (1989–2019), the peak spring counts of GWfG increased significantly with year from less than 2000 individuals in 1989–1991 to over 15,000 individuals in all years after 2008 (except 2013; Fig. 2A; linear regression on log-transformed data, b = 0.032 ± S.E. 0.007, R^2^ = 0.437, F_1,29_ = 22.537, *p* < 0.0001). The peak autumn counts increased slightly from c. 3000 individuals to c. 10,000 individuals between 1989 and 2006 and more sharply from c. 5000 individuals to c. 25,000 individuals between 2007 and 2019 (Fig. 2B; b = 0.024 ± 0.005, R^2^ = 0.432, F_1,29_ = 22.067, p *<* 0.0001). Spring counts rose more rapidly in the early years (1989–2006), whereas autumn counts increased faster more recently (2012–2019) (Fig. 2C).

The break-point regression for the spring counts showed 1997 (± S.E. 4.7 years) as a break-point (R^2^ = 0.476), and the slopes of the relationship indicated faster increase before 1997 (back-transformed slope b = 1.193 ± S.E. 1.106, corresponding to an annual increase of 19%) than after 1997 (1.053 ± 1.027, 5%) (Fig. 2A). For the autumn counts, the break-point was 2011 (± 4.7 years, R^2^ = 0.478), with slower increase before 2011 (1.036 ± 1.019, 4%) than after 2011 (1.141 ± 1.077, 14%) (Fig. 2B).

### Long-term trends in overwintering goose numbers

**Fig. 3:**
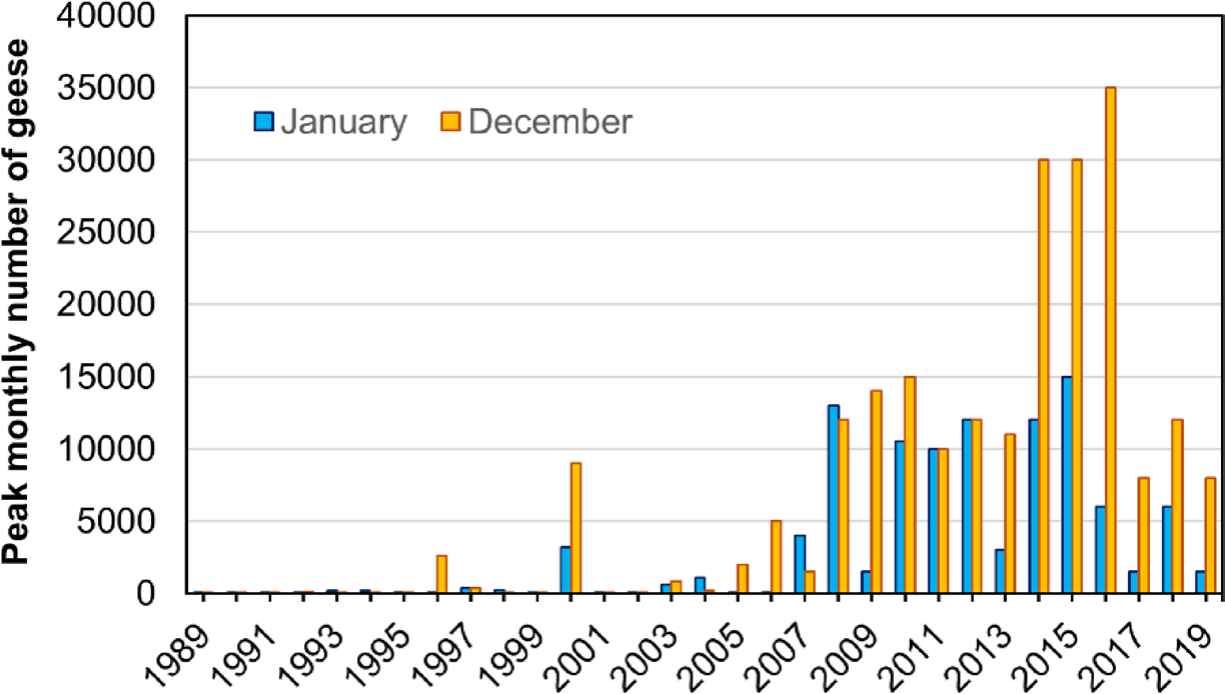
Peak counts of greater white-fronted goose (*Anser albifrons*) in December and the January in the Hortobágy Central Fishponds in 1989–2019.

In years before 2000 (except 1996), most GWfG left the Hortobágy area for wintering sites in W Hungary such as Old Lake at Tata, Lake Balaton and Lake Fertő/Neusiedler See, or in more southerly areas. In years after 2000, an increasing number of geese staging in the autumn migration remained in the study area into December and January (Fig. 3). The peak count of GWfG in December increased from zero to c. 10,000 individuals, and to c. 30,000 individuals in 2014–2016 (Fig. 3). The increase in December peak counts between 1989 and 2019 was highly significant (b = 0.129 ± 0.019; R^2^ = 0.620, *F*_1,29_ = 47.323, *p* < 0.0001). The increase in January peak counts was more moderate but still highly significant (b = 0.108 ± 0.020, R^2^ = 0.510, F_1,29_ = 30.206, *p* < 0.0001).

The increase in January counts was significant both between 1989 and 2012 (b = 0.124 ± 0.032, R^2^ = 0.411, F_1,22_ = 15.376, *p* = 0.0007) and between 2003 and 2012 (b = 0.208 ± 0.075, R^2^ = 0.489, F_1,8_ = 7.644, *p* = 0.025). The rate of change was thus much higher in both periods (c. 12% and c. 21% annually for the two periods, respectively) than the annual rate of increase for the entire flyway population (7.5% and 6.5% for the two periods, respectively^14^).

### Effects of long-term climate change and within-season weather

**Fig. 4:**
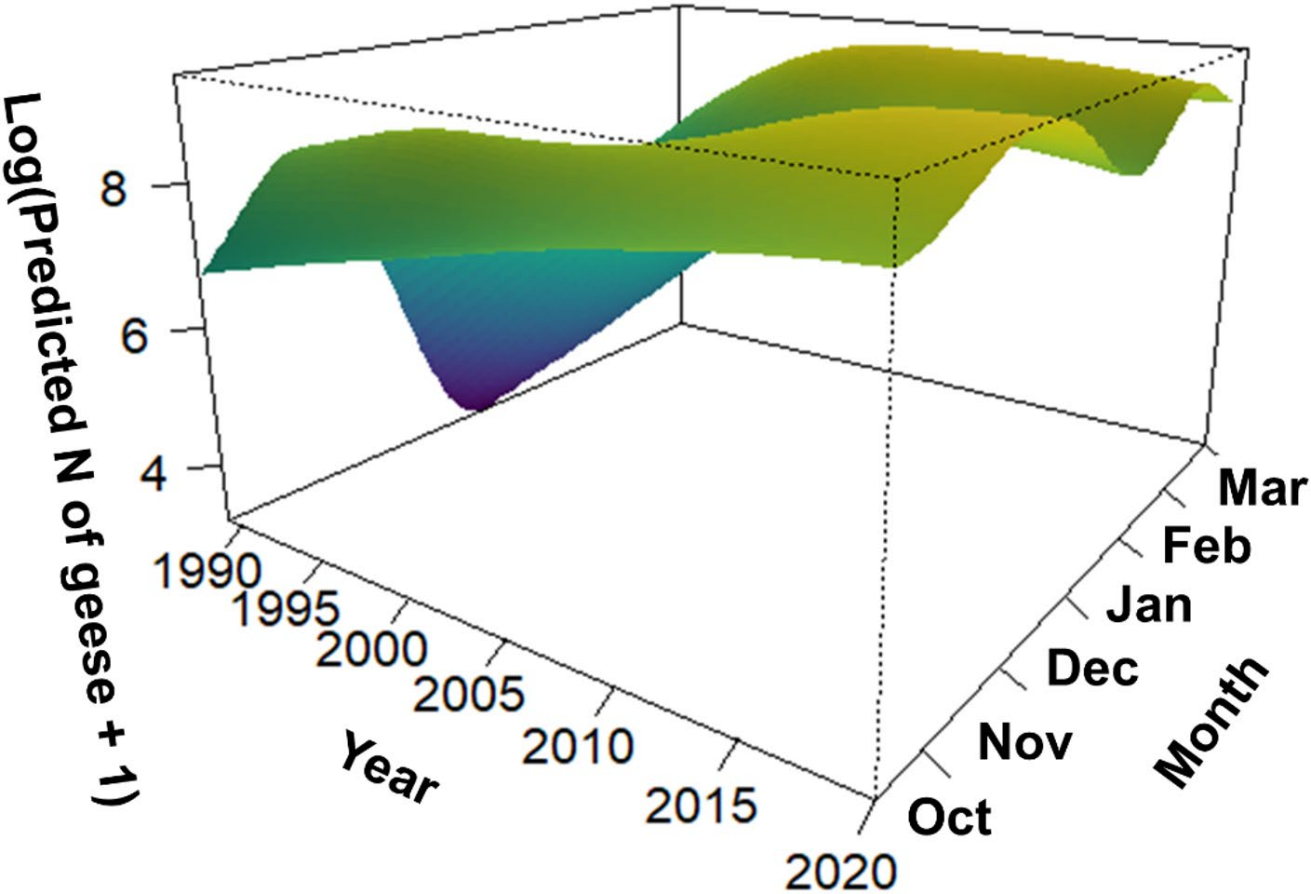
Three-dimensional illustration of long-term (between-year) and short-term (within-year seasonal) effects on counts of greater white-fronted geese (*Anser albifrons*) in the Hortobágy Central Fishponds in 1989–2019, as predicted by the generalized additive mixed model.

The GAMM explained 41.8% of the deviance (adjusted R^2^ = 0.108), with an estimated dispersion parameter of 0.547. The smoothing parameter for the interaction between year and week was highly significant (edf = 11.675, F = 6.979, *p* < 0.0001), indicating strong, non-independent long-term (between-year) and short-term (within-season) variation in goose numbers (Fig. 4). The long-term effect was because of the increase of the stopover population over 31 years, especially in the winter months (Dec-Jan, Fig. 4), whereas the short-term effect was because of seasonal fluctuations, with generally more geese in the autumn (Oct-Nov) and spring (Feb-Mar) than in the winter, albeit with a continuous, long-term increase in the winter counts (Fig. 4).

**Fig. 5:**
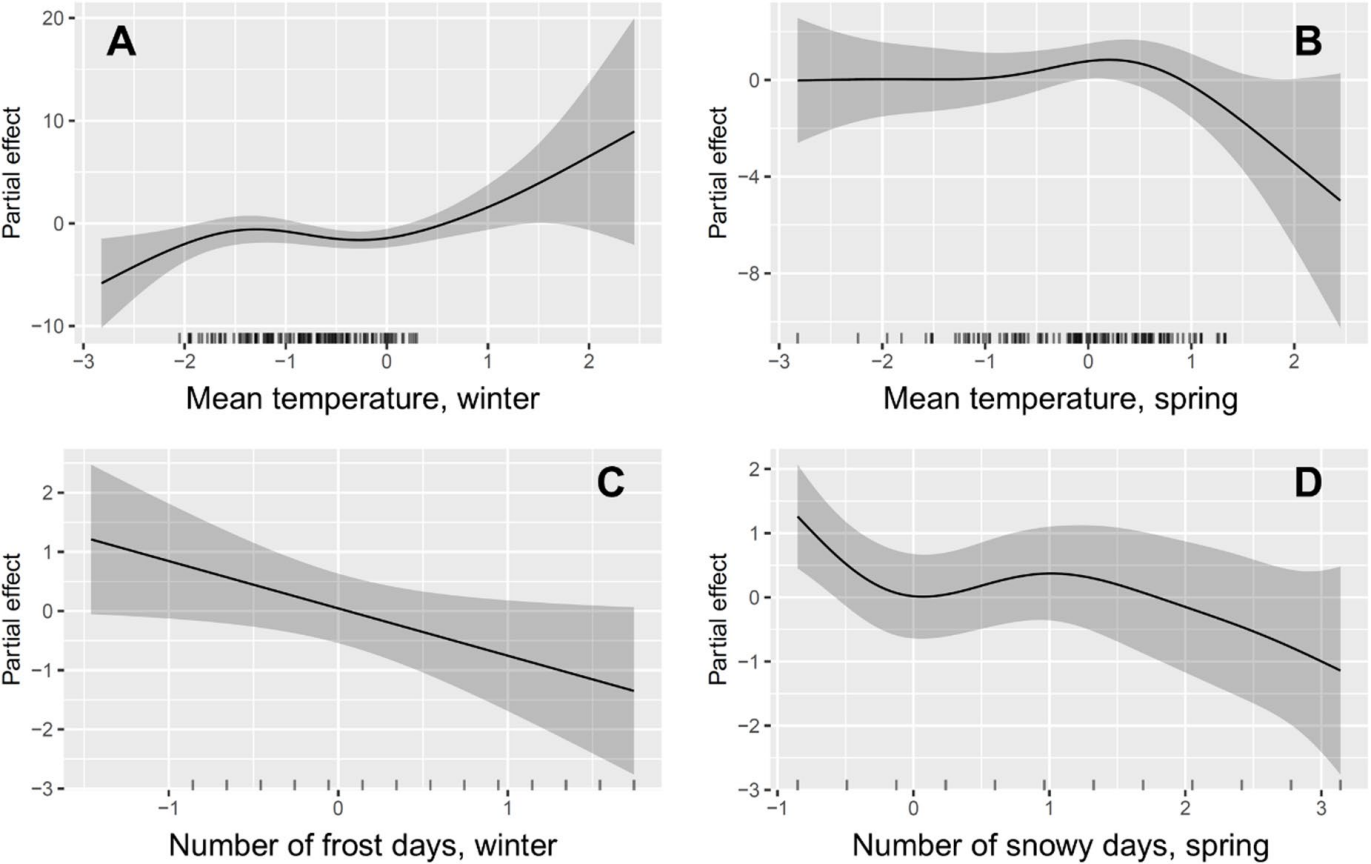
Partial effect relationships predicted by the generalized additive mixed model between goose counts and mean winter temperature (**A**), mean spring temperature (**B**), number of winter frost days (**C**) and number of spring snowy days (**D**).

Mean temperature had no detectable effect in the autumn but was a significant predictor of goose counts in the winter and spring. The relationship was non-linear and positive in the winter, indicating fewer geese in colder weather (Fig. 5A, efd = 3.410, F = 2.766, *p* = 0.020), whereas it was non-linear and negative in the spring, indicating fewer geese in warming weather (Fig. 5B, edf = 3.341, F = 2.728, *p* = 0.030). The number of frost days had a linear negative effect on goose counts in the winter (Fig. 5C, edf = 1.000, F = 4.361, *p* = 0.038), indicating fewer geese with increasing frequency of frost days independently of temperature. The number of snowy days mattered only in the spring, and its effect was non-linear negative (Fig. 5D, edf = 3.380, F = 3.437, *p* = 0.011), indicating fewer geese with increasing snow cover. All smooths for precipitation were non-significant (*p* > 0.05), indicating that precipitation did not influence the goose counts. Finally, the smooth of observation ID was highly significant (edf = 2.957, F = 6.350, *p* = 0.0001), indicating that there was a residual temporal structure not captured by the seasonal tensor, likely consisting of short-scale temporal dependence of observations. The low value of edf (2.957) suggested that there was no overfitting and that the model controlled residual autocorrelation appropriately.

**Fig. 6:**
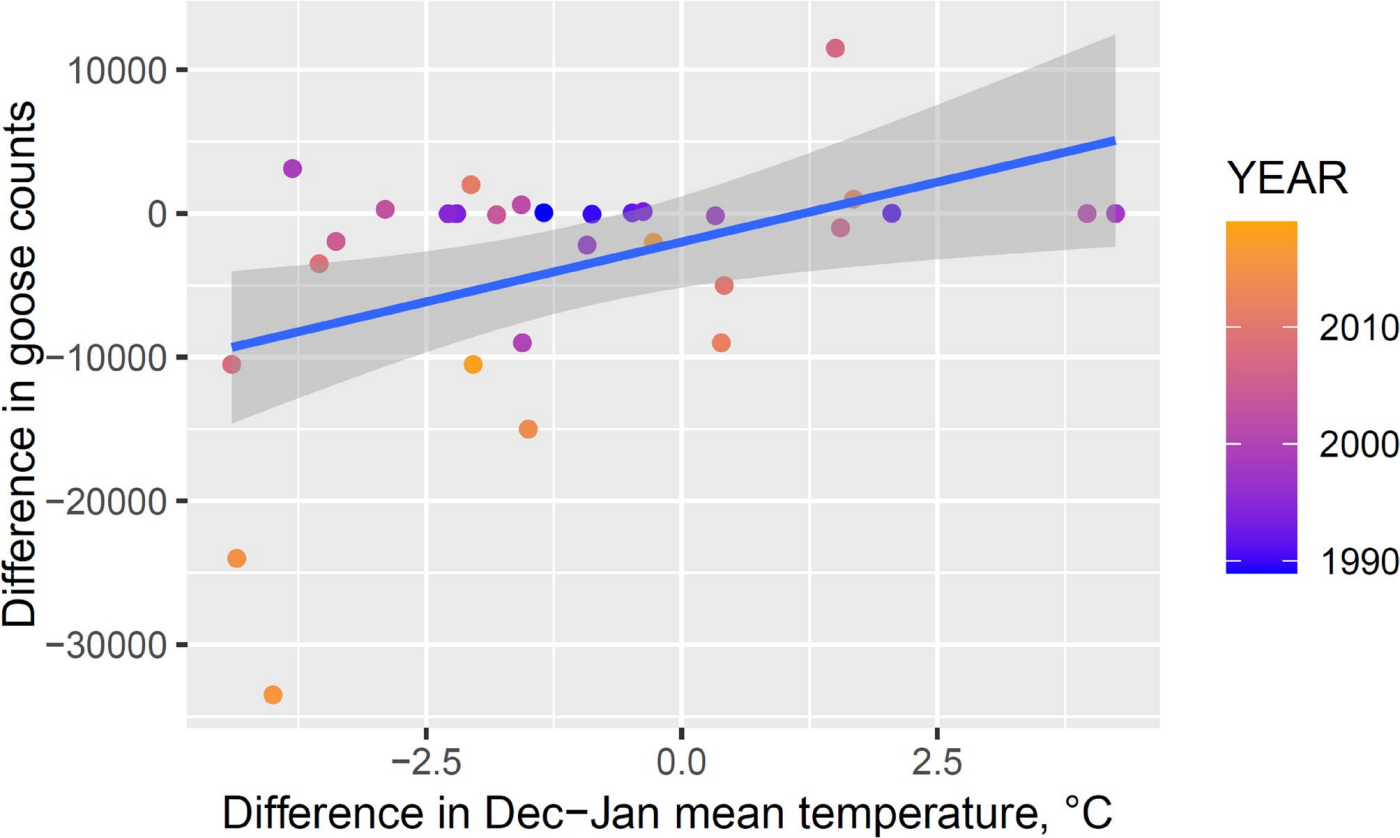
Relationship between the difference in maximum goose counts and in mean temperatures between December and the following January in the Hortobágy Central Fishponds in 1989–2019.

Finally, the difference in GWfG counts between December and the following January was positively related to the difference in mean temperature between these two months (Fig. 6, b = 1666.3 ± 633.30, R^2^ = 0.198, F_1,28_ = 6.922, *p* = 0.014), indicating that the number of GWfG decreased mostly in those winters when mean temperature further decreased from December to January.

### Land cover change in four study periods

**Fig. 7:**
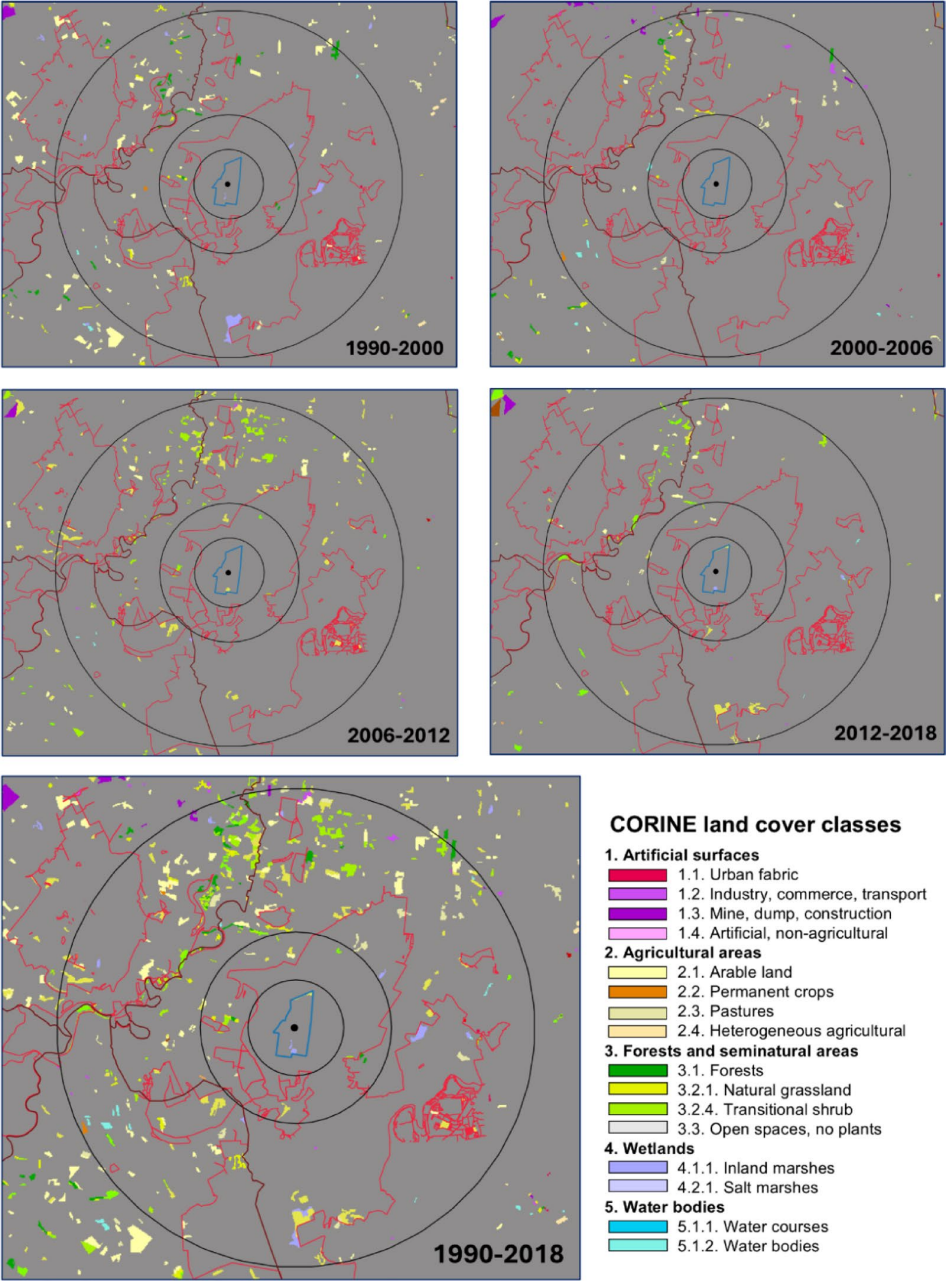
CORINE land cover change maps from the four study periods and from all periods combined. Land cover did not change in areas in grey, while colours other than grey indicate the new land cover. Red lines indicate protected area borders and black lines indicate buffers 5, 10, and 20 km from the Central Fishponds of Hortobágy National Park. The maps were created in QGIS (version 3.40.7) (QGIS Development Team^27^).

In general, there was little change in land cover as changes rarely reached 1% of the area of the buffers around the Central Fishponds in the 28 years between 1990 and 2018 (Table 1). Changes relevant to geese (cropland, grassland, wetland) that exceeded 0.2% of the area of the buffers included the increase in inland marshes and pastures in 2012–2018 in the 5-km buffer, increases of pastures in 2006–2012 and 2012–2018 in the 10-km buffer and increases of inland marshes, non-irrigated arable land in 1990–2000, pastures in 2000–2006, 2006–2012 and 2012–2018 in the 20-km buffer (Fig. 7; Table 1).

**Table 1:** Changes in area and percentage of CORINE land cover classes in the four study periods and in the three buffers of different size from the central fishponds of Hortobágy National Park.

Buffer diameter and area	Land cover class	Time period									
		1990–2000		2000–2006		2006–2012		2012–2018		1990–2018 total	
		Area (ha)	% total	Area (ha)	% total	Area (ha)	% total	Area (ha)	% total	Area (ha)	% total
5 km, 9801 ha	Inland marshes	13	0.133					**31**	**0.316**	**44**	**0.449**
	Pastures					31	0.316	**40**	**0.408**	**71**	**0.724**
10 km, 39 601 ha	Inland marshes	13	0.033					31	0.078	44	0.111
	Non-irrigated arable land	26	0.066					44	0.111	70	0.177
	Pastures	19	0.048	1	0.003	**88**	**0.222**	**104**	**0.263**	**212**	**0.535**
	Water bodies			16	0.040					16	0.040
20 km, 247 506 ha	Inland marshes	**667**	**0.269**			21	0.008	56	0.023	**744**	**0.301**
	Natural grasslands	157	0.063							157	0.063
	Non-irrigated arable land	**1326**	**0.536**	124	0.050	372	0.150	**541**	**0.219**	**2363**	**0.955**
	Pastures	234	0.095	**589**	**0.238**	**2072**	**0.837**	**733**	**0.296**	**3628**	**1.466**
	Water bodies	38	0.015	136	0.055	54	0.022	26	0.011	254	0.103

Only changes relevant to geese (cropland, grassland, wetland) are given and changes exceeding 0.2% of the buffer area are highlighted in Bold.

**Fig. 8:**
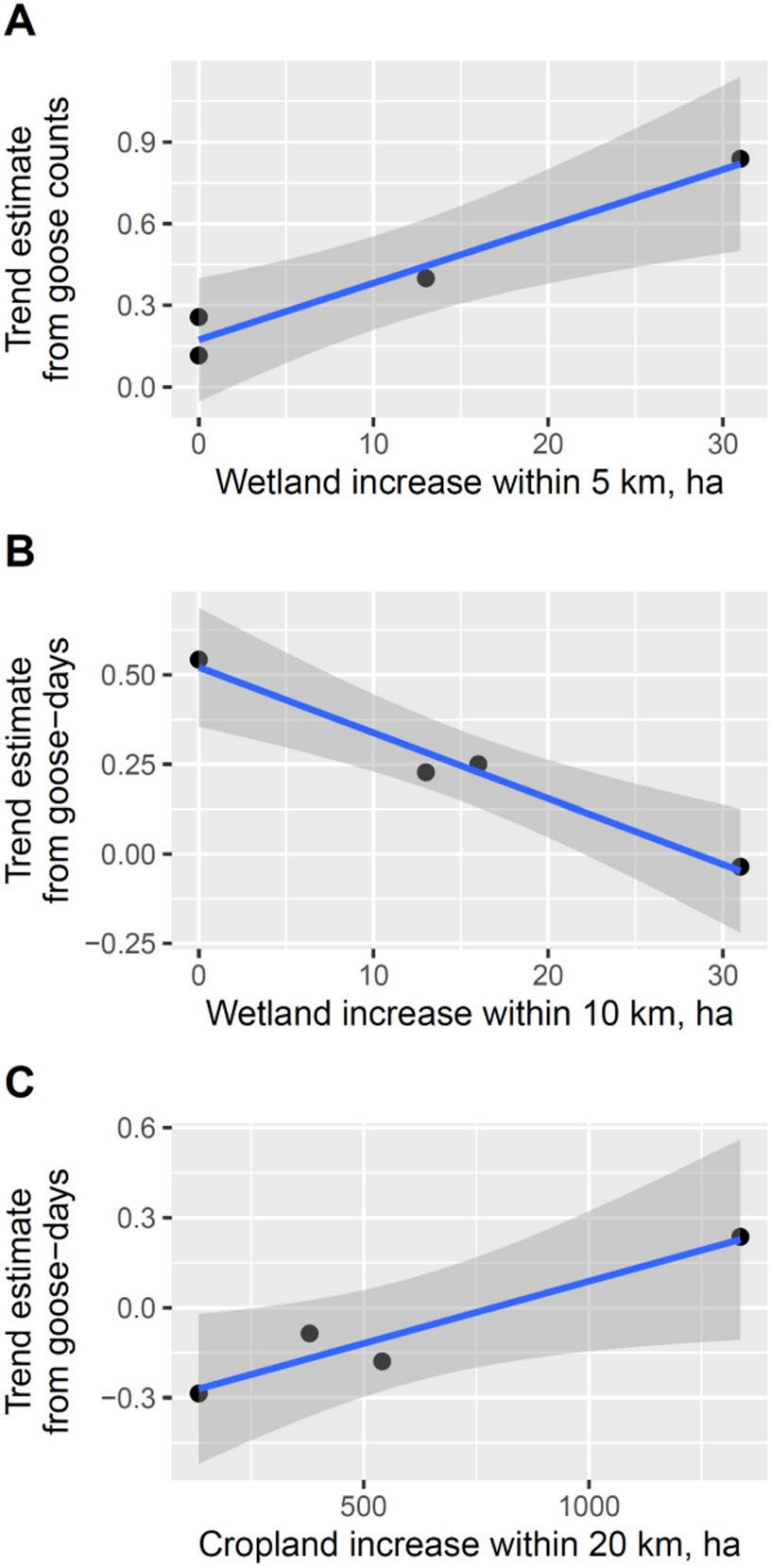
Relationships between trend estimates based on goose counts (**A**) or goose-days (**B**,**C**) and land cover change in buffer areas around the Central Fishponds in Hortobágy in four study periods.

The trend estimate of the migrating population based on autumn goose counts was positively related to the increase in wetland areas 5 km from the Central Fishponds in the four study periods (Fig. 8A, b = 0.021 ± S.E. 0.003, R^2^ = 0.957, F_1,2_ = 44.326, *p* = 0.022). The trend estimate based on the number of goose-days in the autumn was negatively related to the increase in wetland area within 10 km from the Central Fishponds (Fig. 8B, b = -0.018 ± 0.002, R^2^ = 0.975, F_1,2_ = 78.341, *p* = 0.013). Finally, the trend estimate based on the number of goose-days in the spring was positively related to the increase in cropland area within 20 km from the Central Fishponds (Fig. 8C, b = 0.0004 ± 0.000, R^2^ = 0.914, F_1,2_ = 21.292, *p* = 0.044). None of the other 11 regressions of the trend estimate based on goose counts against land cover and none of the other 10 regressions of the trend estimate based on goose-days against land cover change was significant (results not shown).

### Food availability

**Fig. 9:**
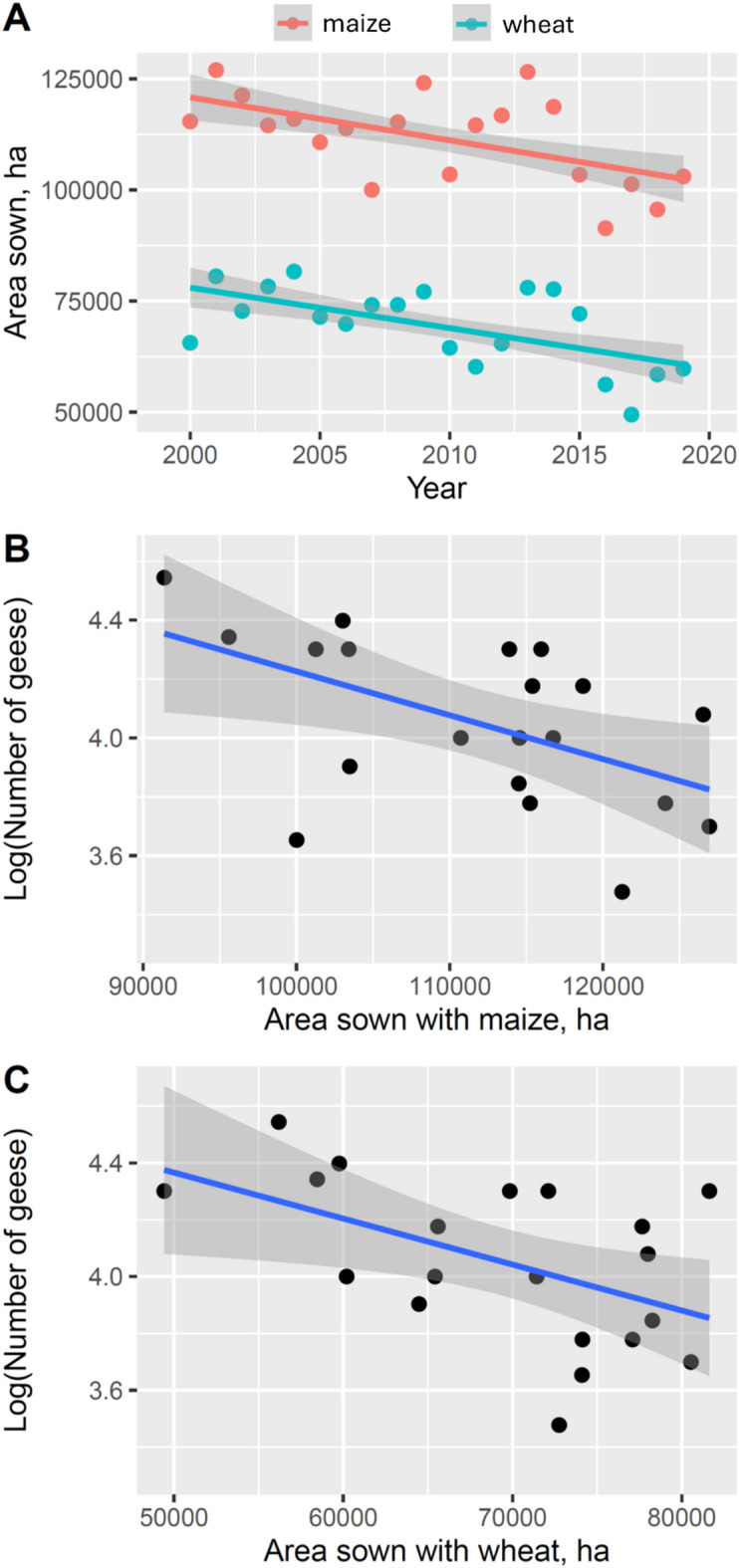
Changes in area sown with maize and wheat in Hajdú-Bihar county between 2000 and 2019 (**A**) and relationship between counts of greater white-fronted geese (*Anser albifrons*) and area sown with maize (**B**) and wheat (**C**) between 2000 and 2019.

The area sown with maize or wheat has decreased significantly between 2000 and 2019 in Hajdú-Bihar county (Fig. 9A, maize: b = -996.7 ± S.E. 335.4, R^2^ = 0.316, F_1,18_ = 8.309, *p* = 0.010; wheat: b = -911.5 ± S.E. 288.1, R^2^ = 0.357, F_1,18_ = 10.008, *p* = 0.005). Neither the number of GWfG counted nor the number of goose-days in the spring were influenced by the area sown with maize or with wheat (linear regressions, n.s.). However, contrary to expectations, GWfG counts in the autumn were negatively influenced both by the area sown with maize and wheat (Fig. 9B, C, maize: b = -0.001 ± 0.000, R2 = 0.279, F_1,18_ = 6.968, *p* = 0.017; wheat: b = -0.001 ± S.E. 0.000, R^2^ = 0.259, F_1,18_ = 6.297, *p* = 0.022). The number of goose-days was not affected by the area sown with maize or wheat (linear regressions, n.s.).

### Disturbance by hunting

**Fig. 10:**
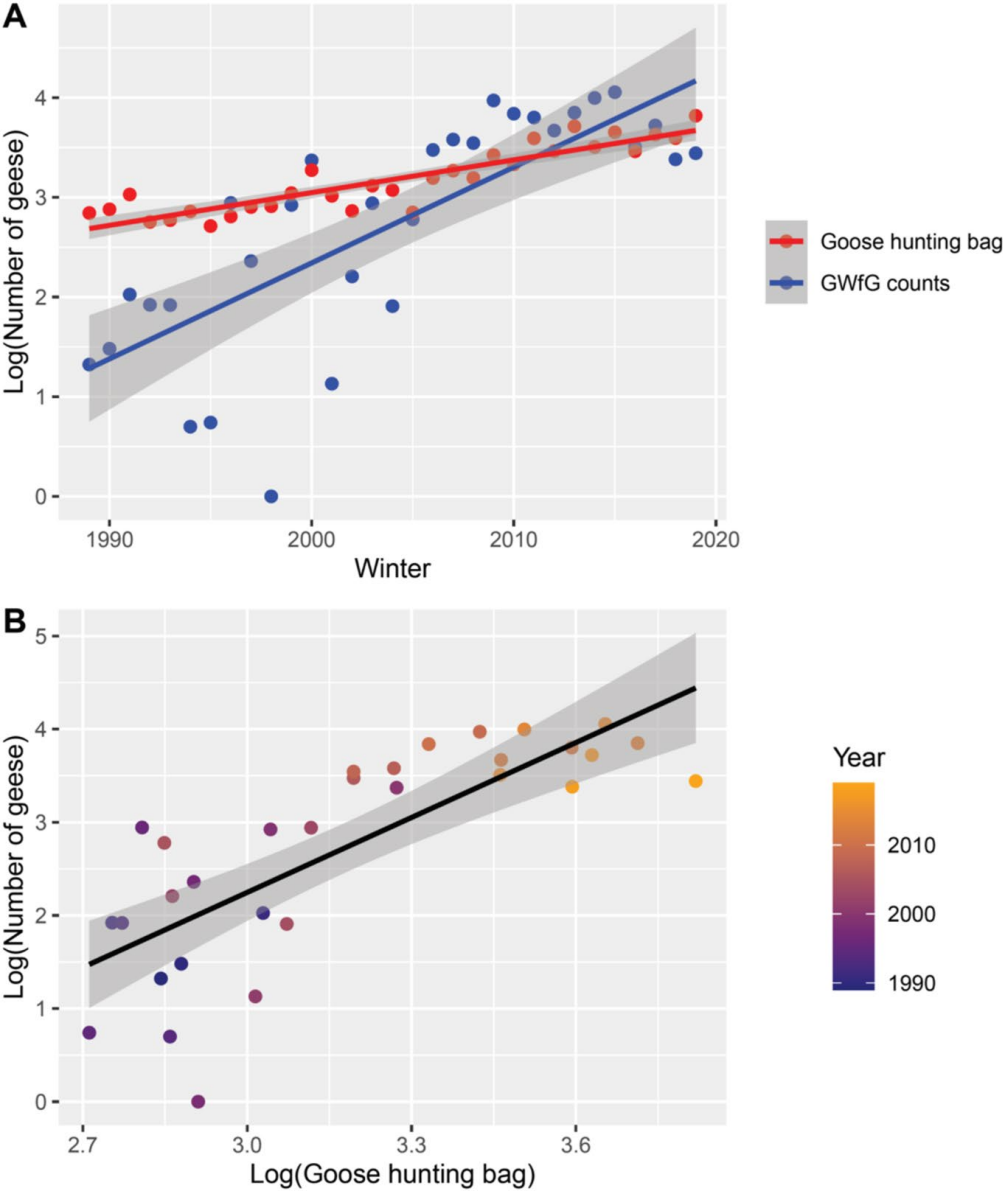
Changes in goose hunting bag in the study county and four neighbouring counties and in counts of greater white-fronted geese (*Anser albifrons*, GWfG) in the winter (December-January, i.e., hunting season) in the study period (**A**), and relationship between goose hunting bag and winter GWfG counts in the study period (1989–2019) (**B**).

Hunting bag data showed that the intensity of goose-hunting in the study county and neighbouring counties increased steadily from few hundred to c. 4000 geese shot per winter during the study period (Fig. 10A). Mean GWfG counts in December and January varied considerably but were generally lower than the hunting bag until the 2005–2006 winter but varied less and were generally higher than the hunting bag beginning in the 2006–2007 winter (Fig. 10A). The rate of increase was much higher for GWfG counts (b = 0.096 ± 0.015, R^2^ = 0.575, F_1,29_ = 41.576, *p* < 0.0001) than for the hunting bag (b = 0.033 ± 0.003, R^2^ = 0.809, F_1,29_ = 128.080, *p* < 0.0001), and the slopes differed significantly (Fig. 10A, interaction F_1,58_ = 17.373, *p* = 0.0001). A direct comparison of hunting intensity and GWfG counts showed that GWfG counts increased as hunting intensity increased (b = 2.682 ± 0.402, F_1,29_ = 44.540, *p* < 0.0001) mostly because of recent (since 2010) years with high winter GWfG counts and goose hunting bags (Fig. 10B). The quadratic term was not significant in any regression model (*p* > 0.09) and the number of goose-days showed patterns very similar to GWfG counts (results not shown).

## Discussion

This study shows that the number of migrating GWfG has increased dramatically in the central Hortobágy region both in the spring and in the autumn since 1989, and that GWfG have started to regularly use the study area for overwintering since c. 2007. By presenting a long-term, 31-year dataset from one of the largest staging and wintering sites of GWfG in eastern Europe, this study is novel in that it fills a gap in our knowledge on GWfG migration and wintering in an understudied part of the range of the species^12^. The increase in GWfG numbers found here generally corresponds with the long-term increase in the total flyway population of GWfG. However, a comparison of change rates in two time periods for which detailed estimates were available (1988–2012, 2003–2012) showed that rate of the local increase (12–21%) greatly exceeded the rate of the increase of the total flyway population (6–8%). This comparison suggested that an increasing proportion of the total flyway population uses the Hortobágy site for staging or overwintering.

Our data also show that the number of GWfG remaining in the study area for winter (December, January) is steadily increasing. The positive relationships between GWfG counts and either winter temperature or December-January temperature differences indicated that warmer weather allows more geese to remain in the stopover site, resulting in higher goose counts. Eventually, this process may have led to the finding that GWfG started to remain in the area for overwintering.

Our results from the period 1989–2007 correspond well with results from the Hungarian Waterfowl Monitoring programme that show that GWfG first occur in the Carpathian Basin in the Hortobágy region on their autumn migration in September^24^. The number of GWfG quickly builds up and peaks here in October and November, then it decreases as GWfG move to other sites in western and southern Hungary and beyond (Germany, the Netherlands) for wintering^24^. The spring migration peak is often much higher, usually above 15,000 individuals in most years (Fig. 2A) than the autumn peak, which reached 15,000 individuals only in four years before 2007 and in all six years since 2014 (Fig. 2B). The spring (February) peak is by far the highest in the entire area of Hungary^24^, which suggests that the Hortobágy area serves as a pre-migration gathering site for the Pannonic flyway population of the species. Our results from the period 2007–2019 corroborate these patterns but also indicate that GWfG started to use the Hortobágy area as wintering grounds. The establishment of new wintering grounds has been observed in several goose species before and has been primarily explained based on climatic changes^2,4,10^, large-scale changes in land use^8^or local management^9^.

Our study shows that climate change fundamentally influenced the number of GWfG that used the Hortobágy area for autumn and spring staging and for overwintering. This was supported by several results. First, the long-term trend showed the largest increase in goose numbers in the winter as opposed to the autumn and the spring months, with no birds in the winter in the early years to regular overwintering by the end of the study period (Figs. 3 and 4). Second, mean temperature influenced goose counts positively in the winter and negatively in the spring (Fig. 5). In winter, warmer temperatures probably resulted in smaller physiological and energetic costs of thermoregulation for GWfG and may even have allowed an increased growth of grasses, the primary food of GWfG. In spring, higher mean temperatures probably induced the continuation of migration to the northern breeding grounds, which likely explains the negative relationship between goose counts and mean temperature. Third, goose counts were negatively influenced by the number of frost days in the winter and by the number of snowy days in the spring, indicating the primary role of sub-zero temperatures and snow in determining goose numbers. Finally, the positive relationship between temperature difference between December and January and GWfG counts between December and January also suggested that more GWfG remained for overwintering when temperatures did not drop or increased from December to January, i.e., when winters were mild.

While the R^2^ value for our GAMM model was relatively low (0.108), such modest values are expected in the context of GAM/GAMM models for ecological count series and should not be overinterpreted^31^. The deviance explained by our model was much higher (42%), and several smooth parameters were significant, which are more important in the context of GAM/GAMM models^31^. A long-term dataset on a staging population of a long-distance migrant bird species such as our dataset typically includes a strong stochastic component and many sources of variation such as annual variability in reproductive success at the breeding grounds, juvenile and adult survival within and between breeding seasons, short-term and long-term population growth rate, food availability at the moulting sites in northern Siberia, annual variability in hunting pressure and other disturbances in previous stopover sites in Russia, Kazakhstan, Ukraine and Romania, variability in the amount of rainfall in the late summer along the route and so on.

Our analyses of alternative, not climate-driven, explanations for the GWfG Pannonic flyway population increase did not find large-scale changes in land cover, identified a long-term decrease in crop food availability and a steady increase in disturbance by hunting in the study region. Land cover changed little in areas close (< 20 km) to the Central Fishponds in the study period (1990 to 2018), and the larger changes (+ 3628 ha pasture, + 2363 ha arable land, + 744 ha inland marshes) occurred scattered in a large area (2475 km^2^) over 28 years (Fig. 7; Table 1). While the trend estimate based on number of geese increased with the increase of wetland area within 5 km from the roosting site, this relationship was opposite (negative) for the trend estimate based on goose-days at the 10-km scale. Considering that the increase in wetland area was quite minimal (0–30 ha depending on the period), these changes are not likely to explain the growth of the migratory and overwintering population of GWfG. Similarly, the increase in cropland area (c. 1300 ha between 1990 and 2000) corresponds to an increase of c. 0.54% of the total 20-km-radius area and thus is unlikely to explain the population growth in this period. Because data on land cover were available in the CORINE CLC database from one period of 10 years and three periods of six years, we cannot exclude the possibility that short-term, i.e., annual, changes in land cover that go undetected in the database contributed to the fluctuation or growth of the Hortobágy staging population of GWfG. Similarly, the CORINE database has a minimum patch size of 25 hectares, and many changes in land cover involving areas smaller than 25 ha can go undetected. Although such short-term or small-scale changes can add up to extensive areas, the areas close (5–10 km) to the study site have been protected as national park where land cover has not changed since the foundation of the park in 1973, thus, the small changes must have occurred farther away, which nevertheless could have still influenced GWfG counts.

The availability of cropland food (maize, wheat), at least based on the area sown with these crops, declined steadily within the study period, and there was also a negative relationship between GWfG counts and the area sown with maize and wheat. The negative relationship is surprising because it suggests that the increase in the staging and overwintering GWfG population is independent of the availability of these resources or that GWfG increasingly use some other key resources, e.g. grasslands for feeding. These results suggest that land cover change or changes in food availability were not likely to explain the large increase in migrating and overwintering numbers of GWfG.

An alternative explanation for the establishment of Hortobágy area as a wintering site can be if former wintering sites in Hungary have become unavailable or suboptimal due to local changes in land cover or management. However, this explanation is unlikely because almost all sites where GWfG are known to winter in Hungary are in protected areas where land cover or management changed little, if any, in the last two decades^35^. Nevertheless, it would be informative to compare December and January counts in the rest of Hungary, neighbouring countries and along the entire flyway with those in the study area to determine how individuals of the species are distributed in relation to weather conditions and food availability in the winter. While Hortobágy is a large staging/stopover site and an emerging wintering site along the path of the Pannonic flyway population, it is still only one of the many sites along the migration corridor. We need regular counts and integrated analysis of the data collected from more sites along the flyway to evaluate the degree and speed of the spatial and temporal redistribution of the flyway population due to climate change.

We cannot fully exclude the possibility that disturbance by hunting played a role in the increase of the overwintering population at the study site. Disturbance by hunting increased steadily during the study period, whereas regular overwintering and the growth of the wintering population started in the mid-2000s (Fig. 3), when the hunting bag of all goose species in the five counties reached c. 1500 individuals per winter (Fig. 10A). While in theory it is possible that this threshold disturbance may have triggered a concentration of wintering GWfG in the Central Fishponds region of Hortobágy National Park, climate effects were probably more important. This is because the geese spending the days outside the park borders probably would have left the study region and the five counties if weather had not been favourable, which likely occurred in winters before the mid-2000s, when hunting was only slightly less intense and GWfG counts varied considerably (Fig. 10A). We also note that causation is likely the other way around: the more geese, the more geese leave the national park for their daily feeding, and the more hunters hunt for them. Taken together, these observations suggest that while the effect of disturbance by hunting cannot be excluded, it probably played a lesser role in explaining the occurrence of overwintering than climate change.

Our study has some limitations that need to be considered in the interpretation of the results. First, our counts likely underestimate the number of GWfG using the study area because flocks may have gone undetected or undercounted, which is a general problem with counting large flocks of birds. We aimed to reduce this bias by conducting two counts daily, one in the morning when geese departed from the roosting fishponds to the feeding areas and one during mid-day when geese returned to the fishponds for mid-day roosting. To minimise undercounting, we took the larger of the two counts for estimating GWfG numbers. Second, we could study the impact of land cover change on GWfG numbers only in four time periods of the 31 years due to limited data availability, resulting in linear regression models based on only four datapoints. While more datapoints may have resulted in a higher number of significant relationships, our data showed that land cover changed little (Fig. 7; Table 1), thus, further datapoints probably would not change our conclusions. Finally, we did not consider the impact of changes in the management of areas within and beyond the national park boundaries. Most grasslands within the national park are grazed, mostly by cattle, or mowed once typically in late June or early July. These practices have not changed substantially in the study period, although the proportion of mowed areas slightly increased due to the decline of livestock grazing. In addition, some conservation management actions resulted in an increase of certain resources for GWfG. For example, more than 1000 hectares of croplands were restored to grasslands in several EU LIFE projects within the park^36^. Grassland quality and food availability on some of the remaining croplands improved due to conservation management conducted to specifically benefit the critically endangered Fennoscandian population of the Lesser White-fronted Goose (*Anser erythropus*) staging in the area (Ecsedi et al. 2009). However, the areas managed in these projects were either small (few tens of ha) or were far (> 10 km) from the fishponds and thus were not likely to explain the large increase in GWfG counts.

In conclusion, climate change appeared to influence the increase of the number of staging and overwintering GWfG while changes in land cover and hunting disturbance probably also contributed. Our study suggests that the increases in the number of GWfG staging and overwintering in the Hortobágy Fishponds, which exceeded the general long-term increase of the total flyway population, are primarily a result of climate change. It is of course possible that density-dependent processes such as increased accumulation of individuals from other, smaller, more crowded staging areas or wintering grounds along the flyway also played a role in this increase (e.g. Layton-Matthews et al. 2019). In summary, our study provides an example for a climate-driven change in the increased use of a staging area and the appearance of a new wintering site, mainly influenced by an increasing frequency of mild weather conditions in the winter months of December and January.

### Conservation implications

Should the increase in numbers continue at the study site, we should consider management options to better accommodate increasing numbers of GWfG within the park area and in croplands around it. In spring, the geese feed preferentially on natural or restored grasslands in Hortobágy, but in autumn and winter they typically feed on croplands. The main cropland feeding sites used by the geese lie outside the border of the national park, where the geese are exposed to greater levels of hunting and more disturbance during the hunting season from 1 December to 31 January. This means that the geese likely experience higher disturbance and increased mortality in December and January than in other times of the year.

One solution to this could be to better manage the extensive existing areas of croplands (mainly maize and wheat fields) as sacrificial goose feeding areas near the roosting sites within Hortobágy National Park. This would potentially greatly extend the existing area of their foraging options, as well as protecting them by reducing their degree of exposure to hunting. Such management would allow the geese to feed without disturbance and could potentially further reduce the turnover time, encouraging the geese to remain even longer within the study area in autumn and into winter.

## Data Availability

The datasets used and analysed during the current study are available from the corresponding author on reasonable request.
